# School life during COVID-19: a qualitative study exploring English secondary school staff and pupils’ experiences of the school-based mitigation measures

**DOI:** 10.1186/s12889-025-21696-6

**Published:** 2025-03-03

**Authors:** Sarah Bell, Jane Williams, Sabi Redwood, Jeremy Horwood

**Affiliations:** 1 The National Institute for Health and Care Research Applied Research Collaboration West (NIHR ARC West) at University Hospitals Bristol and Weston NHS Foundation Trust, Bristol, UK; 2 Population Health Sciences, Bristol Medical School, University of Bristol, Bristol, UK; 3 School of Psychology, University of Bedfordshire, Luton, UK

**Keywords:** Schools, Teachers, Pupils, COVID-19 mitigation measures, Implementation, COVID-19 risk, Safety, Mental health and wellbeing, Qualitative, Public health

## Abstract

**Background:**

In England, the national Government was responsible for balancing the risks of COVID-19 infection, transmission and illness against the known risks of school closures. The Department for Education (DfE) issued guidance to schools, however, there is limited empirical evidence on the experiences of staff and pupils affected by the guidance and accompanying COVID-19 mitigation measures.

**Methods:**

This qualitative study explored secondary school staff and pupils’ views and experiences of COVID-19 guidance and mitigation measures. There were two main objectives: (i) to examine implementation effectiveness, and (ii) to explore their effectiveness at promoting safety. Participants were purposively sampled from English schools serving diverse communities participating in the CoMMinS (COVID-19 Mapping and Mitigation in Schools) study. Semi-structured interviews were conducted remotely, and data were analysed thematically.

**Results:**

Interviews took place between January and August 2021 with participants from five secondary schools (20 staff and 25 pupils); staff represented a range of roles within the school and pupil demographics varied.

Main themes were: (i) negative views of the DfE guidance; (ii) negative experiences of the DfE guidance; (iii) ineffectiveness of the DfE guidance and school mitigation measures at promoting safety and reducing risk; (iv) ineffective implementation of the mitigation measures due to poor adherence and acceptability (with sub-themes for Lateral Flow Testing (LFT), face coverings, physical distancing and ventilation); and (v) positive perceptions (with sub-themes for hygiene measures, and approaches that facilitated implementation and safety which included staff enforcing compliance, having an ethos of co-operation, addressing inconsistencies, and minimising change).

**Conclusions:**

Insights from this research will help understand effectiveness of the measures in the ‘real-world school setting’. Understanding the experiences of staff and pupils will help to support policymakers and school leaders in future pandemic decision-making. This research identified challenges with the guidance and measures, minimal impact on perceived safety, and a negative impact on wellbeing. These challenges should be considered when assessing the benefit of the measures in keeping schools safe.

**Supplementary Information:**

The online version contains supplementary material available at 10.1186/s12889-025-21696-6.

## Background

The UK Health Security Agency (UKHSA, formerly Public Health England) provide best practice guidance [1] and resources [2] for schools on mitigating infectious diseases. The World Health Organisation (WHO) declared the outbreak of COVID-19 as a pandemic on 11 March 2020 [3]. In response, the Department for Education (DfE) issued guidance to UK schools [4–9] that aimed to minimise COVID-19 transmission and support the safe operation of schools to avoid disruption to schooling. It was considered vital for schools to remain open due to the known risks of school closures to education, and to both physical and mental health of pupils [10, 11]. Moreover, school closures are also reported to have a disproportionately negative impact on socio-economically disadvantaged pupils [12].

For schools to operate safely and minimise COVID-19 transmission, they were required to implement mitigation measures and have a ‘live’ risk assessment (continually updated by senior staff). Table 1 lists the mitigation measures outlined in the DfE operational guidance [1]. A timeline with key changes to the measures prior to, during and after the study is provided (Supplementary File 1).

**Table 1:** COVID-19 mitigation measures outlined in the DfE operational guidance for schools

Face coverings (masks, visors)
Ventilation (mechanical i.e. air purifiers and non-mechanical i.e. opening windows and doors)
Hygiene—respiratory and hand (information, increased facilities and practices)
Hygiene—sanitising and cleaning (information, increased practices)
Mass COVID-19 testing (lateral flow testing, LFT), contact tracing, isolation, and shielding
**Physical distancing measures**
Bubbles (keeping the same pupils and staff together)
Restrictions on large gatherings and events (assemblies, parents evenings, concerts, enrichment events)
Staggered lunch times and start and end times to the school day, separate entrance and exit points to school buildings
Restricting movement in classrooms and around school (one-way systems, teachers moving between classrooms opposed to pupils, and a marked out two metre box for teachers to operate from at the front of the classroom)

Gurdasani and colleagues (2022) suggested that UK policy did not give young people sufficient priority and questioned the evidence behind decisions. They stated that schools reopened without sufficient measures to protect them, with flaws highlighted around implementation of the measures. They reported that the DfE failed to update their guidance in response to available data, for example on the importance of airborne spread (and monitoring indoor air quality and providing effective ventilation) [13].

A review in 2020 (updated in 2022) identified and mapped the evidence on school-based COVID-19 mitigation measures [14]. It was noted that 33 of the 38 studies used a mathematical modelling design and only five used real-world data. The urgent need for empirical research assessing the effectiveness of the measures within the school setting was highlighted [14].

A review reported that young people (defined as aged 10 to 24) were less likely to be adversely affected by COVID-19 in relation to their physical health [15]. Young Minds (2020) raised concerns over the negative impact COVID-19 mitigation measures were having on young people’s mental health and wellbeing [16].

Before the guidance was implemented in UK schools, Lorenc and colleagues (2021) used interviews to examine school staff and family views and concerns towards the proposed measures. Pupils, their parents and school staff generally felt the measures were acceptable and pragmatic, but staff had reservations around feasibility and ‘how the measures would play out in practice.’ Negative unintended consequences of the measures on behaviour, learning and pastoral care were anticipated [17]. Before lateral flow testing (LFT) was introduced to UK schools, Denford et al. (2022) explored staff, pupil and parent perspectives using interviews, and concluded LFT may be a feasible and acceptable alternative to isolation [18].

Empirical research to understand actual experiences of the mitigation measures outlined in the DfE guidance to UK schools has been limited. Two studies in UK primary schools (aged 4 to 11 years) reported that staff found implementing and adhering to the measures challenging (both used an online cross-sectional survey; Sundaram et al. (2021) also used semi-structured interviews and Marchant et al. (2021) linked the survey results with routine data on COVID-19 test results) [19, 20].

Kim and colleagues (2020–2022) examined English primary and secondary school teacher experiences at three timepoints during the pandemic. They reported that due to increased job demands as a result of the COVID-19 measures, teacher mental health and wellbeing declined over time [21–24]. Powell and colleagues (2022) conducted a qualitative questionnaire survey with secondary school pupils in England about their perceptions of the pandemic, returning to school, and risk and infection control measures. They reported poor adherence to physical distancing measures (particularly for younger pupils and for all pupils when amongst friends), and to wearing face coverings, but reported testing to be acceptable. They reported that pupils were not anxious about returning to school, and any concerns were mostly about transmitting the virus to family [25].

The aim of this qualitative study was to explore the experiences of English secondary school staff and pupils during the COVID-19 pandemic, to provide empirical evidence of effectiveness of the DfE guidance and mitigation measures within the secondary school setting. Findings will be used to make recommendations to support policymakers, public health practitioners, and senior leaders in schools in future pandemic decision-making. The impact of the mitigation measures on school life and staff and pupil mental health and wellbeing is reported separately.

## Methods

Qualitative interviews were conducted to explore secondary school staff and pupils’ views and experiences of the guidance and school-based mitigation measures. Perceptions of implementation effectiveness and the effectiveness of the mitigation measures at promoting safety were examined.

Staff and pupils were recruited from secondary schools in the South West of England participating in the CoMMinS (COVID-19 Mapping and Mitigation in Schools) study [26]. Participants were purposively sampled to ensure a range of schools, staff roles (e.g. headteachers and teaching assistants) and pupil demographics (e.g. % eligible for Free School Meals (FSM) and age) were included. Semi-structured interviews were conducted remotely (using an online platform or telephone), using interview guides developed for this study (Supplementary File 2) informed by the literature and Government guidance documents.

Staff received the study participant information via the school and were asked to contact the researchers if interested in participating in an interview. Pupils received the study information via a recruitment video and website during a tutor, assembly or Personal Social and Health Education (PSHE) session, or they received the study website link via a school email. Pupils interested in taking part completed an online form. Written parental informed consent was sought for all pupils under 16 alongside verbal assent from the pupil at the start of the interview (digitally recorded); verbal informed consent was sought for all those over 16 years of age and school staff. Pupils were given the option of participating in the interview with their parent/carer or a peer from the same year and school. After the interview, all participants received a £20 voucher to thank them for their participation.

Interviews were digitally recorded, transcribed verbatim, checked for accuracy and anonymised. The transcripts were analysed thematically [27]. Researchers (SB and JW) used line-by-line coding to construct initial draft coding frames, based on three transcripts, using QRS NVivo 12 qualitative data management software [28]. A combination of data-driven inductive and deductive coding, based on the aims of the study and the interview guide, was used. To enhance analysis and maximise rigor researchers (SB and JW) independently double-coded transcripts and discussed discrepancies. The refined coding frame was then applied to all transcripts. This process was completed separately for the staff and pupil data.

## Results

Staff and pupils were recruited from five diverse secondary schools; four mainstream schools and one alternative provision setting. In total, 20 school staff (12 (60%) female) participated including headteachers, senior leaders, class teachers and support staff (between January and July 2021). In total, 25 pupils took part between March and August 2021 (14 (56%) female). Two interviews were paired (with a nominated peer) and one comprised three peers; one interview was supported by a parent and another by an older sibling. Pupils ranged from Year 7–12 (Year 7 n* = 7, Year 8 *n* = 2, Year 9 *n* = 3, Year 10 *n* = 6, Year 11 *n* = 1, Year 12 *n* = 6). Twenty eight percent were receiving FSM**, 44% were white British, and 40% were in the key worker/vulnerable group attending school during lockdown closures. Table 2 provides the sample characteristics.

**Table 2:** Sample characteristics

Sample characteristics	School 1	School 2	School 3	School 4	School 5
n^a^ pupils in school	< 1000	< 1000	< 200	< 1000	> 1000
Year groups (Yr)	Years 7–11	Years 7–11	Years 3–13	Years 7–11	Years 7–13
n^a^ pupils interviewed	5	8	3	1	8
n^a^ staff interviewed	5	3	4	3	5
% eligible for Free School Meals (FSM)^b^	36.5%	12%	77%	34.9%	16.6%
IMD 2019 quintile (within school type)^c^	Most deprived (5)	Least deprived (1)	Most deprived (5)	Next most deprived (4)	Average deprived (3)

^a^n number of participants

^b^FSM proxy measure for deprivation

^c^Index of Multiple Deprivation

Themes from the findings were: (i) negative views of the DfE guidance; (ii) negative experiences of the DfE guidance; (iii) ineffectiveness of the DfE guidance and school mitigation measures at promoting safety and reducing risk; (iv) ineffective implementation of the mitigation measures due to poor adherence and acceptability (with sub-themes for LFT, face coverings, physical distancing and ventilation); and (v) positive perceptions (with sub-themes for hygiene measures and approaches that facilitated implementation and safety).

### Negative views of the DfE guidance

Staff viewed the guidance as inadequate at supporting schools to effectively implement the mitigation measures. It was described as frustrating, impractical and unclear. It was reported to be difficult to follow due to frequent changes and updates, and there was often a lack of time to enable effective implementation. Table 3 provides illustrative quotes.

**Table 3:** Negative views of the DfE guidance

“The stuff from Government was very frustrating in the fact that it was often late and impractical. It changed constantly. A complete lack of understanding of the practicalities of running a school.” (P15, school 1, Headteacher)
“I think obviously with the pandemic, it’s really, really hard to be clear and consistent but other country’s education systems seem to have managed much better than ours.” (P13, school 1, Class Teacher)
“Government changed things so suddenly. Schools had no notice. One thing would be said to us on a Friday, and we’d walk in on a Monday and it would be completely different and it’s because Government had changed the guidelines on a Friday.” (P14, school 1, Teaching Assistant)
“A lot of it was really short notice. Like the new mask thing was literally the night before, we got a message saying that we need to wear masks and that was that.” (P19, school 1, Class Teacher)
“Everything was last minute with the government, they probably should have given schools a bit more autonomy as well.” (P20, school 5, Senior Leader)

### Negative experiences of the DfE guidance

Staff described their experience of using the DfE guidance as stressful and reported feeling burdened by it. The guidance needed to be interpreted and applied in a pragmatic way, with consideration of school context. This responsibility had negative consequences on senior staff who felt burdened continually having to make significant decisions and changes to manage risk. Senior staff also reported feeling burdened by logistical issues. The challenges of timetabling lessons and movement around the building to comply with physical distancing, and issues with space such as being unable to use the canteen or sports hall due to their use as testing sites, were frequently mentioned. These tasks prevented staff from carrying out their usual roles effectively. Staff also identified a financial burden, with no additional resources to implement and adhere to the guidance. For example, significant costs were highlighted to cover staff absence due to COVID-19 infection, isolation or shielding. This had a negative impact on school budgets. For all staff, the guidance resulted in additional tasks and increased demands on their time to manage and deliver the health and safety procedures. Staff expressed how these extra responsibilities were a burden and unrealistic on top of their already challenging workloads. Table 4 provides illustrative quotes.

**Table 4:** Negative experiences of the DfE guidance

“Ultimately what everybody must be aware of is this can go horribly wrong, and if somebody was to contract COVID here, and then heaven forbid it ends in a fatality, the level of responsibility on middle and senior management would be horrendous, and that’s the reality of what we’re working with and the risk we’re trying to weigh up.” (P1, school 3, Senior Leader)
“It is writing policies, procedures, systems, and I keep reviewing them and revising them as the guidelines from the DfE change.” (P18, school 5, Deputy Headteacher)
“The compliance team of the Trust were taking the information and sitting down as a group of people saying ‘Okay, well that’s what they’ve said. Well that’s crazy. We get the point of what they’re trying to do. Right, so how do we interpret that and make it so that we deliver the spirit of it, whatever that was, in a way that’s practical and realistic and meets the needs of the students and the staff.’” (P15, school 1, Headteacher)
“Of course it’s the financial side of things too, it’s put quite a lot of strain on the school’s budget–school budgets in general have been really stretched prior to COVID, as you’re probably aware, then having to buy a whole host of different infrastructure, it’s just clobbered us really.” (P9, school 4, Assistant Headteacher)
“We’re not a health centre. We’ve got a job to do which is big enough at the best of times and then take into consideration the lost learning time and different emotional issues that students have, we can’t really afford to be doing more of the health care side of things. We just haven’t got the time.” (P5, school 3, Senior Leader)
“The lack of preparation time that’s been offered to schools in general has had a really bad knock-on effect with pupils understanding what’s going on, what’s happening and what they need to do.” (P1, school 3, Senior Leader)

### Guidance and measures ineffective at promoting safety and reducing risk

Participants’ accounts suggest that the DfE guidance and related mitigation measures did not effectively promote a sense of safety or reduced risk in school. Many staff and pupils reported feeling unsafe, vulnerable, and at risk of catching and transmitting COVID-19, which was reported to negatively impact their wellbeing. Pupils mostly expressed concern about transmitting COVID-19 to their families. Staff reported a lack of support and acknowledgement by Government of the risks, and many consulted their unions to raise concerns. Feeling unsafe and at risk was primarily attributed to lack of pupil compliance to mitigation measures. Staff working directly with pupils with special education needs and disabilities (SEND), reported increased risk and stress due to additional complications with pupils’ understanding of and adherence to the measures. Fears were often heightened when staff became aware of colleagues being seriously ill following COVID-19 infection, and when cases were high, particularly within their own school. Staff also felt a responsibility to protect the safety of vulnerable staff and there was high anxiety particularly when restrictions were lifted after the first lockdown, with many staff not feeling comfortable returning to school. Table 5 provides illustrative quotes.

**Table 5:** Guidance and measures ineffective at promoting safety and reducing risk

“There was extreme anxiety at the time when we were returning back to school. We had staff who lived with vulnerable people and they were being asked to come back into school. So, some people were anxious about that. They either had vulnerabilities themselves or they had people living with them who were classed as vulnerable and they didn’t want to pass on COVID.” (P16, school 5, Senior Leader)
“We are in a building with a thousand people in it all breathing all over each other and that’s been really scary and lots of staff were very anxious about going back to work, very anxious and had been in touch with the unions just to say you know, ‘where do we stand on all of this stuff?’ I even emailed the head teacher before the last lockdown and I cc’d the union in as well and I said, ‘Actually, school is feeling very dangerous and unsafe’.” (P12, school 1, Class Teacher)
“I had lots of conversations with staff, like, ‘I don’t feel safe in this room currently because there’s someone right next to me’ and the site team and Senior Leadership Team had said, ‘We’ve checked the rooms’. And they had checked the rooms but then, you know, a kid moves a table.” (P6, school 2, Senior Leader)
“I felt that it was a risk, I was in twice a week. We were in bubbles by this point, but I just felt like it was a risk that I really didn’t want to have to take.” (P20, school 5, Senior Leader)
“Some of the pupils just haven’t come in because they’ve been too worried. About three or four have been having nightmares, sleep loss, they’re scared to go out.” (P14, school 1, Teaching Assistant)
“Some pupils were just really worried about coronavirus and taking it home to their parents.” (P7, school 3, Senior Leader)
“What kind of viruses do they have if they don’t wear a mask? There was no real punishment for not wearing a mask.” (Pupil 13, school 1, Yr 7)
“Whenever there was a COVID case you were worried that you could then get it.” (Pupil 15, school 4, Yr 10)

### Implementation of mitigation measures ineffective

Both staff and pupils perceived implementation of the mitigation measures, except hygiene practices, to be ineffective due to poor acceptability and adherence. Personal choice not to comply, deliberate rule breaking, lack of understanding, misunderstanding, or simply forgetting to follow the rules were reported.

P12, school 1, Class Teacher, “When you’re in the flow of teaching– when you’re in the zone and in the flow – that kind of self-consciousness evaporates and you’re just doing what you’re doing and it’s suddenly like, ‘Oh s**t I’m too close to you or I’m not masked up.’”

P4, school 2, Senior Leader, *“There are some parents who don’t want their children to wear a mask or get vaccinated or even do the test. I can think of a student who doesn’t wear a mask, doesn’t have the test and he hasn’t even taken the home test home, nothing.”*

P18, school 5, Deputy Headteacher, *“There’s a limit to what we can do. We have heard of students who are meant to be self- isolating all down in Cabot Circus, all hanging out together having a little party, but what can we do? We enforce to the parents in our communication that they have a duty to keep their child at home. A huge number of our students don’t live around the school area, they live substantially further away and so just how are we supposed to enforce that?”*

A summary of the negative views and experiences of each mitigation measures follows, with illustrative quotes.

#### Lateral flow testing

Staff found LFT a burden on their time. Validity and reliability concerns were reported, and acceptability of the procedure was poor which resulted in low testing uptake. In addition, a number of pupils discussed misleading information around testing from social media, the news, and from friends and family, that may have impacted compliance. In some schools, where parents did not speak English as their first language, there were additional barriers to conveying information about testing. Isolation procedures (having to isolate after being identified as a close contact of a positive COVID-19 case) were also viewed negatively by staff and pupils. They were often described as being inconvenient and disruptive to teaching and learning, and to pupil wellbeing due to a negative impact on them socially. Table 6 provides illustrative quotes.

**Table 6:** Lateral flow testing

*“Something that kind of panicked staff and kids is that the lateral flow tests are only 40 percent effective so yes, there’s still 60 percent chance that you could be walking round with it and not knowing. And our first case in school that we had was somebody that had no symptoms at all. It was just because he got a track and trace text and then he went and got a test and he came back positive. So that worried the wellbeing of some people I think understandably.”* (P7, school 3, Senior Leader)
*“Staff run the testing and it was phenomenal hours. Like it would take three or four staff members half a day nearly to do that for 50 people.”* (P5, school 3, Senior Leader)
*“We have a number of agency covers that have to come. With increased COVID rates and teachers having to self-isolate, it does have an impact on the whole school.”* (P16, school 5, Senior Leader)
*“I heard that we only had about a 60% uptake in terms of parents consenting to it, you just think it undermines the entire system if 40% of the kids are going round the school untested – anyway, that’s political.”* (P12, school 1, Class Teacher)
*“We report to the school and we’re supposed to report to the NHS app as well but I must admit I haven’t been doing the NHS app if I’ve been negative, I must admit. I do them obviously if it’s positive but when it’s negative—I can’t access it on my phone at home, you see, so I do it at home and then I think, ‘Oh, I’ve got to remember to log it next day with the school.’ So sometimes I do three at once [laughs], you know, backdate it.”* (P19, school 5, Class Teacher)
*“I know I’ve missed doing it a few times because I’ve been late getting up and going to bed so I haven’t had time to do it and when I didn’t, the school haven’t emailed or anything to say you haven’t done.”* (Pupil 3, school 2, Yr 8)
*“It’s kind of just annoying because obviously I want to keep checking if I have corona or not but we’re expected to do a school day and do an hour of exercise a day and do homework and do a home testing kit and they take like half an hour to fully develop and yes, it’s a lot.”* (Pupil 6, school 2, Yr 9)
*“The uptake for the lateral flow tests wasn’t great, I mean nobody really likes sticking anything up their nose or in the back of their throat, there hasn’t been a massive uptake of that.”* (P3, school 3, Senior Leader)
*“Every week in tutor or every two weeks we get the Covid tests, but they make me feel so sick after, so I only make myself do one once a week. We’re supposed to do it three times a week but I can’t do it.”* (Pupil 8, school 2, Yr 7)
*“Parents thought they were being asked to have their children injected and it was like, ‘no, no, no, it’s a lateral flow test’. I think as well because of the nature and the demographic of our school that language barriers have been an issue.”* (P12, school 1, Class Teacher)
*“I get scared that the swab is going to get stuck in there and that I’ll have to get it taken out, I’m scared of that. And there’s a lot of fake news going around that apparently the swabs have cancer and stuff. It just doesn’t make sense, but a lot of people in my class are like ‘oh no, my mum doesn’t want me to get it because it gives you cancer’.”* (Pupil 7, school 2, Yr 7)

#### Face coverings

There were many factors contributing to poor adherence to and acceptability of wearing face coverings. They had a negative impact on communication, and teaching and learning. Inconsistent practice of the need to wear face coverings sometimes resulted in apathy around use. There were also frequent reports from pupils of them being uncomfortable (described as hot, rough, itchy, tickly, sweaty, hurting their ears and causing spots). Table 7 provides illustrative quotes.

**Table 7:** Face coverings

*“I’ve complained quite a lot because the bacteria from my mask, I’ve gotten a lot of spots around my mouth and my cheeks and my nose and they’ve really started to hurt because I’m wearing this mask all day and I can't take it off.” (*Pupil 3, school 2, Yr 8)
*“I have really bad acne sometimes, wearing the masks are just uncomfortable. It made me itch, I had to keep pulling it off every two minutes. In class, it was just horrible you could barely drink water because everyone would be looking at you, ‘Why is your mask not on?’”* (Pupil 21, school 5, Yr 12)
*“It’s a bit rough. It takes my make up off, it’s horrible.”* (Pupil 2, school 3, Yr 12)
*“In that first week back compliance was very good and the message was, ‘Look folks, this is the deal that we can be back in the building, that we all wear these retched things’ and then inevitably over the course of the three weeks we were back things start slipping.”* (P12, school 1, Class Teacher)
*“A lot of the students walking around the school, they don’t wear them and if they do wear them, they don’t wear them properly.”* (P14, school 1, Teaching Assistant)
*“People were just taking them off, they only wore it when they saw teachers around.”* (Pupil 21, school 5, Yr 12)

#### Physical distancing measures

Physical distancing measures were considered the least acceptable due to being the most challenging to implement, adhere to, and most disruptive to school life. They aimed to manage movement in and around the school, in addition to who pupils were allowed to mix with. These measures often lacked consistency, such as living with a sibling but not being allowed to mix with them at lunchtime. This led to frustration and indifference which appeared to contribute to poor adherence and acceptability. Rules around physical distancing measures were reported to not make sense. Staff and pupils often expressed frustration about the lack of clear instructions, information and support from senior leaders, for example on how to navigate the one-way systems around the building. Physical distancing measures significantly disrupted school routines and social contacts and staff and pupils reported feeling restricted, a sense of uncertainty and of loss. Schools with less space had greater difficulties implementing them, as did staff working with pupils with SEND. There was also a lack of belief in their effectiveness at reducing risk of COVID-19. Many teachers reported struggling to adhere to physical distancing measures and unable to teach and manage their pupils’ behaviour effectively due to them. Table 8 provides illustrative quotes.

**Table 8:** Physical distancing measures

*“I think it’s a bit annoying because we have bubbles but our seating plan isn’t with our bubbles. So if I caught COVID I would give it to everyone who sits near me and my whole bubble so that’s like half the class gone so I don’t really understand it.”* (Pupil 5, school 2, Yr 7)
*“We didn’t have a clear instruction of which stairs were ‘up’ stairs and which stairs were ‘down’ stairs and so we just had kids crossing on the stairways. I know for a fact that kids are walking up and down the stairs that I use and we’re all together on those stairs going up and down and there’s no social distancing.”* (P10, school 1, Class Teacher)
*“The kids were breaking their bubbles, they were being yelled at for breaking their bubbles but they just did because they’re kids. They don’t understand the consequences to any massive degree. ‘I wanna go see my friend over there, I’m gonna go walk over the playground and see my friend over there’. It happened all over the place so it felt like a massive amount of effort for very small returns on the reduction in the potential rate of infection.”* (P15, school 1, Headteacher)
*“We are expected to stay in our box at the front of the classroom. Now I cannot do behaviour management and stay in my box at the front of the classroom. It’s not possible. I’m not supposed to leave my box, I’m not supposed to move round the classroom but I do because I cannot teach in the environment that we’re in right now, I cannot teach. I am not sane at the end of a lesson without moving around with the kids.”* (P10, school 1, Class Teacher)
*“You crouch down to their level, working very close to them. You’re definitely sharing equipment. Our style of working hasn’t changed at all really. I mean, we’ve been told to adapt our style of working and we’ve asked how we do it—and they basically say, ‘Well, you know, we don’t know.’ They see us with the students; they know that we’re close to them, they know we’re not wearing masks, they know we’re touching them.”* (P14, school 1, Teaching Assistant)
*“We are in year groups in separate areas, but there are some students who just wander round the school, not following the rules, it just means if they were positive they would be spreading between every year group which is what the whole system is trying to avoid.”* (P13, school 1, Class Teacher)
*“They have to sit in the same seating plan in every lesson so that we can more easily identify well ‘who were they sat next to?’ ‘Who are their close contacts?’ And obviously if they had a different seating plan every lesson that would be really difficult. So that’s impacted on peer relationships because if they particularly get on with the person that they’re sat next to or if they get on okay with them, but they’re not like their best friend, they’ve had much less opportunity to develop new friendships because they’re in their year group bubble, they’ve sat next to the same people for every lesson so it had impacted on.”* (P17, school 5, Senior Leader)
*“It’s eating up a lot of people’s time, by staggering their break and lunches for example, you have the senior leadership team essentially on lunch duty for an hour and a half every day which is a huge chunk of time that could be better used.”* (P9, school 4, Assistant Headteacher)
*“They were going in the other bubbles, they were mixing and it wasn’t right but the teachers saw and didn’t do anything, which I thought was quite wrong, to say the least.”* (Pupil 21, school 5, Yr 12)
*“They tried to keep us in our bubbles. But to be honest, keeping us in groups of six did not work at all. It impacted people’s mental health.”* (Pupil 8, school 2, Yr 7)

#### Ventilation

Schools relied on non-mechanical ventilation (i.e. opening doors and windows) opposed to air purifying monitors, due to high costs and lack of their availability. Opening windows was reported to be disruptive due to insects coming into class, outside noise disrupting lessons, and cold temperatures in class. Opening internal doors blocked the view of the whiteboard for some pupils which impacted learning, and propping doors open often broke fire regulations. In some cases, ventilating was difficult as windows had been painted shut. Pragmatic decisions were made which often negatively impacted adherence. Table 9 provides illustrative quotes.

**Table 9:** Ventilation poor acceptability and adherence

*“I bet you would see in the news that children were freezing and this and that…it’s not ideal, so you just close the windows more, it’s not perfect.”* (P8, school 4, Assistant Headteacher)
*“The difficulty with us and because our building is so old –the windows are so old that they don’t open – there are things that the school can’t do in terms of ventilation.”* (P4, school 2, Senior Leader)
*“Obviously keeping all the windows open is okay at this time of year but during the winter was obviously more of a challenge, well not a challenge– it’s not difficult to do but it’s difficult to put up with the complaints of, ‘It’s cold in here sir.”’* (P9, school 4 Assistant Headteacher)
*“It depends where you are in the school, how well ventilated the classrooms are. The room I’m in at the moment is quite well ventilated, it’s up high, the wind comes through, but some classrooms literally have the sunlight all day into them and they only have windows that open, like, that much.”* (P19, school 5, Class Teacher)
*“The teachers do prefer the windows to be open which is fine now that it's summer, but in the winter we also have vents on the ceiling, which half the time never stay closed. And having the windows open and the doors open, it was always really cold. So, most people were wearing their coats and getting in trouble for it.”* (Pupil 15, school 4, Yr10)

### Positive perceptions

Findings conveyed negative perceptions of the guidance and mitigation measures. Although limited, some positive perceptions were shared by staff and pupils.

#### Hygiene measures

Hygiene measures (cleaning and sanitising and respiratory and hand hygiene) were acceptable to both staff and pupils, as these were reported to be easy to implement and adhere to. Table 10 provides illustrative quotes.

**Table 10:** Hygiene good acceptability and adherence

*“The handwashing hasn’t been a problem massively really. The kids have been okay to do it. They have preferred using antibacterial gel which I know isn’t as effective but it’s better than nothing. So yes, they’ve been okay with that actually. They’ve not been too bad.”* (P7, school 3, Senior Leader)
*“Hand sanitiser is more convenient than soap and water because it’s just on hand so quickly, you know on every corridor there’s at least one on those hand sanitiser stations. Yeah it’s just quick and easy really.”* (P9, school 4, Assistant Headteacher)
*“Pupils are generally okay with washing hands and hand gel. Yes that’s become second nature and I think it has for all of us in a way.”* (P11, school 4, Pastoral Support Teacher)
*“Spraying down the tables, it’s not that big a deal.”* (P10, school 1, Class Teacher)
*“They’ve got into the routine of wiping down their Chromebooks and lapbooks and putting them back in the trollies.”* (P11, school 4, Pastoral Support Teacher)
*“I have a system in my classroom, we have this big blue roll that we use so when I’ve got a spare minute I just sit and rip sheets off it so they’re all ready to hand out and then I always elect or actually ask for volunteers and the kids are dying to do it.”* (P12, school 1, Class Teacher)
*“We fog our building every night, the children are cleaning stuff down, there’s hand sanitiser around in every room and we’re paying overtime to our cleaners. I’ve got two cleaners, bless them, who just spend all their time wandering around wiping surfaces. I joked to them and said ‘You must have worn away the bannisters by now’.”* (P15, school 1, Headteacher)
*“We stopped staff doing things like sharing cups and cutlery, we said bring in your own, take it home with you.”* (P2, school 2, Headteacher)
*“You can’t just take in a basketball and then hand it out to somebody in a different year groups so we have to do things like spraying it down, put them in different bags – but, yeah you get used to it after a while, it was a bit of a pain at first but, you know, it just became one of those things. The lunchtime supervisor would do that so there was a huge amount of adaptation to people’s roles and a lot of goodwill involved as well.”* (P9, school 4, Assistant Headteacher)
*“They brought in a new system with the bells when there’s a transition between period one and two and then period three and four and then between five and six. There’s a warning bells that goes which is a three minute before the lesson ends warning bell which is to remind you to be packed up by then and that the last three minutes of the lesson is dedicated to cleaning and sorting.”* (P12, school 1, Class Teacher)
*“I don’t really mind it to be honest. It’s my own priority to sanitise my hands so I just do it before I eat and wash my hands before I eat.”* (Pupil 6, school 2, Yr 9)
*“When we get to school every morning we get met at our entrance by our head of year and pastoral manager they make sure as everyone walks passed they're okay, make sure they hand sanitise.”* (Pupil 15, school 4, Yr 10)

Despite hygiene measures having the best adherence and acceptability, a few pupils did report sore hands from increased handwashing and sanitising.

Pupil 13, school 1, Yr 7, *“Well, with handwashing – you wash your hands so often, your hands start flaking, and it becomes really sore.”*

Senior leaders also commented on the additional financial burden of extra cleaning and sanitising.

P7, school 3, Senior Leader, *“We’ve had to buy-in an extra cleaner to be able to cover the amounts that they’re meant to be cleaning and the times that they’re meant to have been cleaning for as well.”*

#### Approaches that facilitated implementation and safety

Staff enforcing mitigation measures and their associated rules in person (in corridors, classrooms and exits and entrance points to the school) and through policies, for example by adding face coverings to the uniform policy or rules to the behaviour policy, facilitated implementation and perceived safety. Some schools reported taking a strict, zero-tolerance approach to non-compliance as being most effective. Higher adherence and acceptability were reported in schools encouraging an ethos of cooperation and a positive culture, as this resulted in greater acceptance of social and school norms. Senior leaders operating an ‘open door approach’ about safety and wellbeing concerns for staff and pupils resulted in the school community feeling listened to and valued. This also ensured individual needs were responded to. Implementing approaches such as actively addressing inconsistencies in procedures and messaging and potentially misleading information (often from external sources such as social media) was beneficial. As was providing unambiguous instructions and clear messaging. Other supportive approaches included reducing changes to rules and disruption to daily school life as much as possible and allowing sufficient time for measures to be embedded. Finally creating and adopting innovative implementation ideas was often a success. One example was a school using technology to enable vulnerable staff with subject expertise to teach from home when schools reopened, whilst an adult in school supervised the behaviour and management of the class. Table 11 provides illustrative quotes.

**Table 11:** Approaches that facilitated implementation and safety

**Enforcing rules and strict zero-tolerance approaches**
*“Since the second lockdown, since we’ve come back just before Easter, the pupils are very good, and we’ve got very strict measures in place.”* (P11, school 4, Pastoral Support Teacher)
*“We even went as far as saying we would use the behaviour policy and exclude for a temporary exclusion if students wouldn’t comply and we did have to use that a couple of times.”* (P9, school 4, Assistant Headteacher)
*“We’ve taken quite a hard line on it …there’s just not time in the day or the energy to be expended in the day having arguments about wearing face masks, it’s just a zero tolerance. All the students have to wear a face mask and we did adapt our uniform policy to include face masks being worn.”* (P9, school 4, Assistant Headteacher)
*“Everybody’s on it, the consistency has been a rule of thumb, it’s like let’s just hammer them with this. So in tutor time every morning you know, it’s like, ‘Masks on, masks on’ and before we leave classrooms.”* (P12, school 1, Class Teacher)
*“As soon as we walked in we had to get our temperature and sanitise and every so often, sanitise and wash our hands. The school was strict on it.”* (Pupil 9, school 3, Yr 12)
*“We’re supposed to issue detentions for people who don’t have their masks.”* (P19, school 5, Class Teacher)
**Having an ethos of cooperation and positive culture**
*“There’s a real sense of collective endeavour, camaraderie perhaps, that is quite palpable and is a positive thing.”* (P2, school 2, Headteacher)
*“Everybody has a sense of ‘how can we do this and how can we achieve this for our students and ourselves’, it’s a bit like you know in the war time they had all that sort of spirit, well we’ve kind of got that same spirit I suppose.”* (P11, school 4, Pastoral Support Teacher)
*“The staff are very good like that, I think people realised it was exceptional circumstances and needs must, you just have to crack on really. The commitment has been brilliant. Absolutely brilliant. But not sustainable.”* (P9 school 4, Assistant Headteacher)
*“It’s definitely brought people together to find solutions.”* (P6, school 2, Class Teacher)
*“Staff have been really good about it. I can only praise our staff about how they’ve pulled together and just no-one’s complained.”* (P18, school 5, Deputy Headteacher)
“Just keeping a positive vibe, yes and recognising the hard work people are doing.” (P5, school 3, Senior Leader)
**Addressing inconsistencies**
*“It’s really difficult to explain inconsistencies to teenagers. Teenagers don’t like − in fact, teenagers love identifying inconsistencies. In a school you need consistency, that’s what works for them. They find it really difficult that, ‘it’s okay for me to sit next to this person now, but when I’m at break time you’re telling me I have to stay a distance away from my friends? What is the point in that?’ And that’s difficult to explain to them. We try to turn it around and talk about, whenever you can, you always make the safest possible choice, and by doing that you make our community safer. Try to change the conversation…”* (P2, school 2, Headteacher)
**Having an ‘open-door policy’**
*“Our headteacher very much has an open-door policy. If you’re struggling, you go and see her.”* (P11, school 4, Pastoral Support Teacher)
*“The message was really clear to us as well that actually if you don’t feel safe being back in this building, you need to be in touch with your line manager and a conversation needs to be had about that.”* (P12, school 1, Class Teacher)
**Minimising change**
*“I think one of the hardest things for staff is that constant change. We’ve tried really hard to say, ‘this is what we’re doing this term, we’re not changing it’. And even sometimes when you’ve gone, ‘oh, gosh, I wish we hadn’t − I wish we weren’t doing it like that,’ we’ve actually stuck to it because we’ve just thought the best thing is not to change.”* (P2, school 2, Headteacher)
**Creating and adopting innovative implementation ideas**
*“Staff that were classed as clinically vulnerable, once we had the technologies up to speed and once training was there, they’ve taught with cover supervisors observing the class, so that’s been an interesting step forward. So those teachers teach from their home, on the big screen in their classroom, whilst an adult oversees behaviour, so it does mean the subject specialism has continued, to be able to offer that expertise.”* (P8, school 4, Assistant Headteacher)
*“People talk about how amazing these vaccines are, what science has delivered. But teachers have innovated, have done years of innovation in weeks.”* (P2, school 2, Headteacher)

## Discussion

This qualitative study reports the experiences of COVID-19 mitigation measures from the perspective of English secondary school staff and pupils. Participants reported negative views and experiences of the DfE guidance and mitigation measures. Staff felt significant burden due to additional health and safety responsibilities and increased workload. The guidance and measures were limited in promoting a sense of safety and reduced risk for staff and pupils and were found to have a negative impact on their wellbeing. Implementation of the mitigation measures, except hygiene practices, was ineffective due to poor acceptability and adherence. Physical distancing measures were considered the least acceptable due to being the most challenging to implement, adhere to, and the most disruptive to school life. Hygiene measures were most accepted as these were easy to implement, adhere to, and the least disruptive. Approaches that facilitated implementation included enforcing compliance, having an ethos of co-operation, addressing inconsistencies, and minimising change.

Findings from this study provide an understanding of the challenges UK secondary schools faced with implementing the DfE guidance and associated school mitigation measures. Importantly, the measures were not implemented well nor found to improve perceived safety in staff or pupils, and they may have negatively impacted the wellbeing of the school community. These insights will facilitate the response of decision-makers considering use of school-based mitigation measures in future pandemics. They will also help understand effectiveness of the measures in the ‘real-world’ school setting to inform any future policy.

### Implications of the findings in context of other literature

Findings from our empirical research support those of Gurdasani and colleagues (2022) [13]. School staff and pupils did not feel supported by Government and there were many issues implementing the measures. Findings also concur with the studies conducted in UK primary schools [19, 20], and secondary schools [25], that highlighted difficulties with implementation of and adherence to the school-based measures. Denford et al. (2022) [18] predicted LFT to be feasible and acceptable in schools, however staff and pupils in our study did not report this to be the case in practice. Our findings verified staff reservations about feasibility of the measures [17] and findings of increased job demands on staff causing burden and negatively impacting wellbeing [21–24]. Finally, our research supported findings that pupils’ main risk concern was transmitting the virus to their family [25].

A recent review of unintended consequences of mitigation measures implemented in the school setting highlighted 17 qualitative studies from several different countries [29]. Our study focused on UK secondary school staff and pupil experiences of the DfE guidance and measures, and findings were shown to concur with other qualitative studies from the UK [17, 19–25]. However, negative perceptions of COVID-19 guidance and mitigation measures were also reflected in many of the qualitative studies from other countries in this review, despite differences in the course of the pandemic and mitigation measures implemented.

### Recommendations

Timely and unambiguous guidance to schools from Government is recommended in future pandemics, to provide clarity and to minimise uncertainty. Staff expressed the need for minimal changes and disruption to school life and the importance of allowing time for the measures to be embedded. Schools also need additional support and resources to alleviate burden implementing any guidance and measures.

Cleaning and sanitising, alongside hand and respiratory hygiene measures, were found to be easy to implement, adhere to, and acceptable to most staff and pupils, therefore should remain standard practice in schools to prevent and control infections. Opening windows and doors to maintain good ventilation was disruptive, but there is good evidence for mechanical ventilation at reducing transmission risk [30]. All classrooms being fitted with these devices should also be suggested as standard practice in schools, with funding allocated for this.

Face coverings could be enforced in communal school spaces in future pandemics, for example when moving around the school in corridors or on school transport. During the school day when pupils are mixing with those in other year groups (e.g. whole-school assemblies) or when in close contact with others (e.g. a teaching assistant working 1:1 with a pupil), wearing face coverings should be recommended. However, removing them in classrooms to aid communication and teaching and learning would be pragmatic, and this would also minimise any issues with discomfort.

Uptake of testing was poor (potentially missing asymptomatic cases), and isolation had a negative impact on education and wellbeing. Significant staff burden (time away from other activities, logistical difficulties) also resulted from this measure. In agreement with Denford et al. (2022), if testing is deemed necessary in schools in future pandemics, further work is needed to: dispel misleading information about testing; provide clarity on how to conduct the test; and provide information on evidence of effectiveness at testing reducing risk of infection [18].

Physical distancing measures are not recommended by the UK HSS as usual practice in schools to mitigate against infection [1], but were outlined in the DfE guidance [4]. They should be avoided, if possible, due to their poor acceptability and adherence and significant negative impact on both staff and pupil wellbeing.

To increase effectiveness of pupil compliance, evidence-based findings that focus on how measures help reduce transmission of infection to their family should be considered.

Finally, a number of useful approaches that improved implementation effectiveness were identified that could be encouraged such as: staff enforcing compliance, having an ethos around school of co-operation, addressing inconsistencies, and minimising change.

Table 12 lists recommendations from the findings to support decision-makers in future pandemics.

**Table 12:** Recommendations to support policymakers, public health practitioners and school leaders in future pandemics

1. Decision-makers need empirical research on evidence of effectiveness of school-based measures at reducing infection, transmission and illness, to justify their need to the school community. Use of natural experiment opportunities would be desirable
2. The school community need improved information and education around infection, risk and effectiveness of specific measures
3. Government should provide timely guidance and resources to schools, to ensure effective implementation of the measures and to reduce burden on staff
4. When decision-makers are considering implementation of specific mitigation measures, it is important to balance their benefit at reducing risk of infection, transmission and illness, against any negative consequences of the measures on the school community
5. School leaders could use the approaches identified that facilitate implementation of the mitigation measures. Schools should be encouraged to share effective approaches with other schools
6. Schools need autonomy to be pragmatic to meet the needs of their school context. They should be encouraged to be innovative and try alternative approaches to avoid risk (such as use of technology, outdoor education, and smaller class sizes) [31]

### Limitations of the study

The discussions of effectiveness in this study reflect participants’ perceptions. Future studies that include data on the outcomes of mitigation strategies could provide important context for the perceptions reported in this study. Pupil views and experiences of the DfE guidance were limited, and conclusions were drawn mostly from the staff data. Despite this, their negative views and experiences of the measures indicated issues with the DfE guidance. Although the sample size is relatively small, negative experiences of the guidance and measures corroborate with other UK studies [17, 19–25] and studies from other countries [29]. The dynamic COVID-19 situation meant that throughout the study the DfE guidance changed and the evidence-base evolved. Longitudinal data to understanding the impact of evolving guidance on implementation over time would have been beneficial.

## Conclusions

Insights from this research contribute to understanding effectiveness of the measures in the ‘real world school setting’. Findings will aid policymakers, public health practitioners, and school leaders when making decisions in future pandemics. Assessing any benefit of the measures in keeping schools safe from infection, against the negative consequences of mitigation measures on the school community is crucial. Perspectives of those impacted by the guidance and measures should be used to inform any future guidance. Findings from this research have provided evidence of strategies to support schools to facilitate implementation of mitigation measures in the future and highlighted the feasibility and acceptability of hygiene measures as usual practice. Despite young people being deemed less at risk of COVID-19, school staff are also part of the school community and must be protected, alongside pupils’ families in future pandemics. Ambiguities in communication, policy and practice inconsistencies, and misleading information from external sources were found to be damaging to the safety and wellbeing of pupils and staff. Policymakers, public health practitioners and school leaders are advised to work together to achieve clarity and consistency within school and the wider community. It is clear that the school community felt vulnerable during the pandemic. The significant negative impact of the guidance and mitigation measures on staff and pupils calls for schools to be an urgent public health priority to support them to recover.

## Supplementary Information

Supplementary Material 1. Timeline of key changes to the mitigation measures.

Supplementary Material 2. Interview guides.

## Data Availability

The data underlying this article will be shared on reasonable request to the corresponding author.

## Abbreviations

CoMMinS: COVID-19 Mapping and Mitigation in Schools study
DfE: Department for Education
FSM: Free School Meals
IMD: Index of Multiple Deprivation
NHS: National Health Service
PHE: Public Health England
PSHE: Personal, Social and Health Education
SEND: Special Educational Needs and Disability
LFT: Lateral Flow Test
UK: United Kingdom
UK HSA: UK Health Security Agency (formerly Public Health England, PHE)
WHO: World Health Organisation
