# Supplementary material for: School life during COVID-19: a qualitative study exploring English secondary school staff and pupils’ experiences of the school-based mitigation measures
Source: BMC Public Health. 2025 Mar 3;25:845. doi: 10.1186/s12889-025-21696-6 (PMC11877837; doi:10.1186/s12889-025-21696-6)
Supplement: Supplementary file 1 — Supplementary Material 1. Timeline of key changes to the mitigation measures. [file 12889_2025_21696_MOESM1_ESM.docx]

# Supplementary File 1. Timeline of key changes to the mitigation measures

| **Date** | **Mitigation measures** |
| --- | --- |
| 23^rd^ March 2020 | National lockdown and schools closed except for key worker and vulnerable children.  Blended learning. |
| 15^th^ June 2020 | Secondary schools reopen to Year 10 and 12. |
| 3^rd^ Sep 2020 | Secondary schools reopen for all pupils. |
| 5^th^ Nov-2^nd^ Dec 2020 | National lockdown but schools remain open.  Face coverings mandatory in communal areas in school and on transport.  Some physical distancing measures recommended e.g. bubbles.  Hand and respiratory hygiene, ventilation recommendations. |
| **4^th^ January 2021** | Secondary schools closed except for key worker and vulnerable children.  Blended learning. |
| **8^th^ March 2021** | Secondary schools reopen for all pupils.  Face coverings mandatory in all areas in school (including in classrooms during lessons) and on transport.  Physical distancing measures, hand and respiratory hygiene, ventilation recommendations.  Twice weekly LFT for staff and pupils (as well as testing before returning to school). |
| **17^th^ May 2021** | Relaxation of mitigation measures.  Face coverings no longer required.  All extra-curricular activities resume. |
| **July-August 2021** | Removal of some mitigation measures in school.  Bubbles no longer required, gatherings such as assemblies allowed and normal lunch mixing.  Face coverings no longer required in classrooms, communal areas or on transport.    Remaining mitigation measures:  Hand and respiratory hygiene, cleaning and sanitising schedule, ventilation  Continue following public health advice on testing, self-isolation and managing confirmed cases of COVID-19.  Twice weekly asymptomatic LFT for staff and pupils (as well as testing before returning to school).  NHS Test and Trace now responsible for contact tracing. From 16^th^ Aug under 18’s no longer required to self-isolate but advised to take PCR (polymerase chain reaction) test. |
| Autumn term 2021 (Sep-Dec) | Schools implement mitigation measures to suit school context and in line with outbreak management plan with advice from local health protection team.  Vaccination programme for 12 year olds and older rolled out.  From 14^th^ Dec 2021 if identified as contact of someone with COVID-19 strongly advised to take a LFT every day for 7 days and continue to attend school as normal, unless they have a positive test result. |
| January 2022 | Relaxation of mitigation measures.  Bubbles no longer required and mixing e.g. assemblies, lunch resumed.  From 4^th^ Jan face coverings were recommended in classrooms, communal areas and on transport but not for teachers when teaching from front of classroom. Transparent face coverings suggested.  Remaining control measures:  Hygiene, cleaning and sanitising, ventilation recommendations.  Public health advice on testing, self-isolation, manging confirmed cases of COVID-19.  Vaccination programme for 12 year olds and older. |

*Bold indicates period when interviews for this study took place
